# Effect of ethanol in carbon monoxide poisoning and delayed neurologic sequelae: A prospective observational study

**DOI:** 10.1371/journal.pone.0245265

**Published:** 2021-01-11

**Authors:** Sungwoo Choi, Sangsoo Han, Sangun Nah, Young Hwan Lee, Young Soon Cho, Hoon Lim, Myeong Sik Kim, Gi Woon Kim

**Affiliations:** Department of Emergency Medicine, Soonchunhyang University Bucheon Hospital, Bucheon-si, Gyeonggi-do, Republic of Korea; Kaohsuing Medical University Hospital, TAIWAN

## Abstract

**Objectives:**

Carbon monoxide (CO) is one of the most common poisoning substances, which causes mortality and morbidity worldwide. Delayed neurologic sequelae (DNS) have been reported to occur from several days to months after exposure to CO. Thus, there is a need for prevention, recognition, and treatment of DNS. Patients with CO poisoning as a component of intentional suicide often also consume ethanol, but there is debate regarding its role in DNS. We explored whether ethanol has a neuroprotective effect in CO poisoning.

**Methods:**

This prospective observational study included patients who visited the emergency department from August 2016 to August 2019 due to CO poisoning. After treatment of acute CO poisoning, patients were interviewed by telephone to ascertain whether DNS had occurred within 2 weeks, 1 month, and 3 months from the time of CO exposure.

**Results:**

During the study period, 171 patients were enrolled. 28 patients (16.37%) developed DNS. The initial Glasgow Coma Scale (GCS) scores were 15 (10.5–15) for the non-DNS group and 10 (7–15) for the DNS group (p = 0.002). The ethanol levels were 11.01 ± 17.58 mg/dL and 1.49 ± 2.63 mg/dL for each group (p < 0.001). In multivariate logistic regression analysis, the GCS score had an odds ratio of 0.770 (p < 0.001) and the ethanol level had 0.882 (p < 0.030) for onset of DNS.

**Conclusions:**

Higher ethanol level and higher initial GCS score were associated with lower incidence of DNS. Ethanol could have a neuroprotective effect on the occurrence of DNS in CO poisoning patients.

## Introduction

Carbon monoxide (CO) is one of the most common poisoning substances, which causes mortality and morbidity worldwide. It is a major contributor to deaths from poisoning in the United States [1]. Approximately 21,000 people visit emergency departments each year with accidental poisoning; 15,000 annually present with intentional CO poisoning from suicide attempts [2, 3].

Symptoms caused by CO poisoning range from mild (e.g., headache, nausea, and dizziness) to severe (e.g., loss of consciousness, cognitive dysfunction, and death). The brain and heart are the organs most vulnerable to CO poisoning [4]. Delayed neurologic sequelae (DNS) have been reported to occur from several days to months after exposure to CO; they can cause personality changes, psychosis, mild cognitive impairment to severe dementia, and unconsciousness [5, 6]. Recent studies have shown that DNS occurs within 6 weeks in most patients [7, 8], and another study reported the risk is most prominent in the first 2 weeks and remains significant up to 6 months later [9]. DNS exhibit profound effects on the quality of life of patients and their families; thus, the prevention and treatment of DNS are important in patients with CO poisoning [10].

Nearly 50% of CO poisoning suicide attempts involve exposure to CO in combination with other substances, which must be considered during treatment [11]. Among them, ethanol is the most common [11]. Ethanol reportedly has a neuroprotective effect in patients with traumatic brain injury (TBI) [12, 13]. Ethanol is known to reduces leukocytes infiltration and microglia activation, resulting in faster recovery after TBI. The cytokine shifting was induced by the decrease of granulocyte-macrophage colony-stimulating factor, interleukin-3, 6, and the increase of interleukin-13 and vascular endothelial growth factor [14]. It also has a beneficial outcome by affecting the degree of cytokine induction and microglial activation through the signal transducer and activator of transcription 6 pathway [15]. But there is debate regarding its effect in CO poisoning.

One study showed that ethanol had a neuroprotective effect and led to a better neurological prognosis in patients with CO poisoning [16]. Another study reported that ethanol was not neuroprotective; however, it showed a difference in severity and outcome according to the initial Glasgow Coma Scale (GCS) score, regardless of ethanol consumption [3]. Here, we investigated whether ethanol has a neuroprotective effect against DNS in patients who attended the emergency department due to CO poisoning.

## Materials and methods

### Ethics approval

The study protocol was approved by Soonchunhyang University Bucheon hospital Institutional Review Board (No. 2020-03-019-002). All patients gave their written informed consent for study before they were enrolled in the study.

### Patient enrollment and study setting

This was a prospective observational study of patients who visited the emergency department of an urban university hospital (with 60,000 patient visits annually) from August 2016 to August 2019 due to CO poisoning. The enrollment criteria were presentation to the emergency department with acute CO poisoning during the study period. The diagnosis of acute CO poisoning was determined according to the history, physical examination, clinical situation, and carboxyhemoglobin (COHb) level (> 5% for non-smokers and > 10% for smokers). Exclusion criteria were age < 18 years, a history of stroke or head trauma, discharge against medical advice, no serum ethanol measurement data, and incomplete medical records. Patients lost to follow-up (i.e., those for whom we were unable to confirm DNS) were also excluded from the final enrollment.

Hyperbaric oxygen therapy (HBOT) was provided to patients who showed neurological symptoms (e.g., loss of consciousness, seizure), as well as patients with COHb level ≥ 25%, regardless of symptoms. The HBOT protocol was followed as recommended in a previous study [17]. In total, 3 sessions of HBOT were conducted within 24 hours from the time of CO exposure. The first session provided 100% oxygen at 3 and then 2 atmospheres absolute, followed by 100% oxygen at 2 atmospheres absolute for the second and third sessions. Patients without indications for HBOT received normobaric oxygen therapy using 100% oxygen through a non-rebreather face mask; depending on subsequent symptoms, the need for HBOT was evaluated.

### Data collection

Our institution established a CO registry in August 2016 containing the following information: past history, physical examination, GCS score, vital signs, exposure duration, suicidal intentionality, and laboratory results (e.g., COHb, ethanol level, blood cell count, cardiac enzyme levels, and lactate level). This registry was reviewed by two senior emergency medical residents.

Discharged patients who agreed to undergo follow-up were checked by telephone interview to determine whether DNS had occurred within 2 weeks, 1 month, and 3 months from the time of CO exposure, by two emergency physicians who were blinded to the study objective [9, 18, 19]. If the patient reported the symptoms of DNS during the telephone interview, he or she was asked to come to the hospital to be further evaluated for the diagnosis of DNS. The patient was admitted to the hospital, and neurology and psychiatry consultations with brain MRI were performed. Neurologic examinations evaluating the mental status, cranial nerve, motor and sensory function were performed [20]. And for the evaluation of cognitive disorder, a neurologist physician performed Mini Mental State Examination and determined that a score of less than 24 had cognitive disorder [21]. Also, to evaluate psychological disorder, intentional CO poisoning patients were consulted to a psychiatrist at the initial acute phase to evaluate suicidal thoughts and depressed mood. During the follow-up period, if the revisited patients complained depression, a psychiatrist re-consulted the patient to discriminate whether this was a worsening of the previous depression or new symptoms by DNS. The criteria of the Diagnostic and Statistical Manual of Mental Disorders-5 was used. The final diagnosis of DNS was made by the neurologist after excluding other possible causes of neurologic symptoms via history taking, neurologic exam and brain MRI finding [19].

The exposure time was obtained by history taking from the patient. For the patient with unclear consciousness, the information was obtained from paramedics or caregivers and possible maximum exposure time was estimated. Additionally, to determine the degree of ethanol elimination, the time from ethanol intake to the time of blood sampling was recorded. To assess the occurrence of DNS, information regarding various symptoms (e.g., depression, cognitive abnormality, difficult concentrating, lethargy, mutism, emotional change, amnesia, psychosis, gait or movement abnormality, apraxia, and agnosia) was obtained from the patient or patient’s caregiver by using a predetermined script and list of questions (questionnaire is presented in supporting information section). Since there is not yet an established diagnostic scale for DNS, if any of the above symptoms were seen, MRI was performed and a neurologic consultation was conducted, and then DNS was finally diagnosed.

### Statistical analyses

The distributions of continuous variables were investigated using the Shapiro–Wilk test and a histogram; these variables were analyzed using Student’s t-test and the Mann-Whitney U test. Categorical variables were analyzed using the chi-squared test or Fisher’s exact test. Odds ratios and 95% confidence intervals were calculated using a multivariate logistic model. A multivariate logistic regression model was created using the results of univariate analyses. In the multivariate logistic regression model, creatinine kinase, CO exposure time, GCS score, and ethanol level were analyzed. Statistical analyses were performed using IBM SPSS Statistics, ver. 26.0 (IBM Corp., Armonk, NY, USA) and R, ver. 3.5.3 (R Foundation for Statistical Computing, Vienna, Austria).

## Results

**Fig 1:**
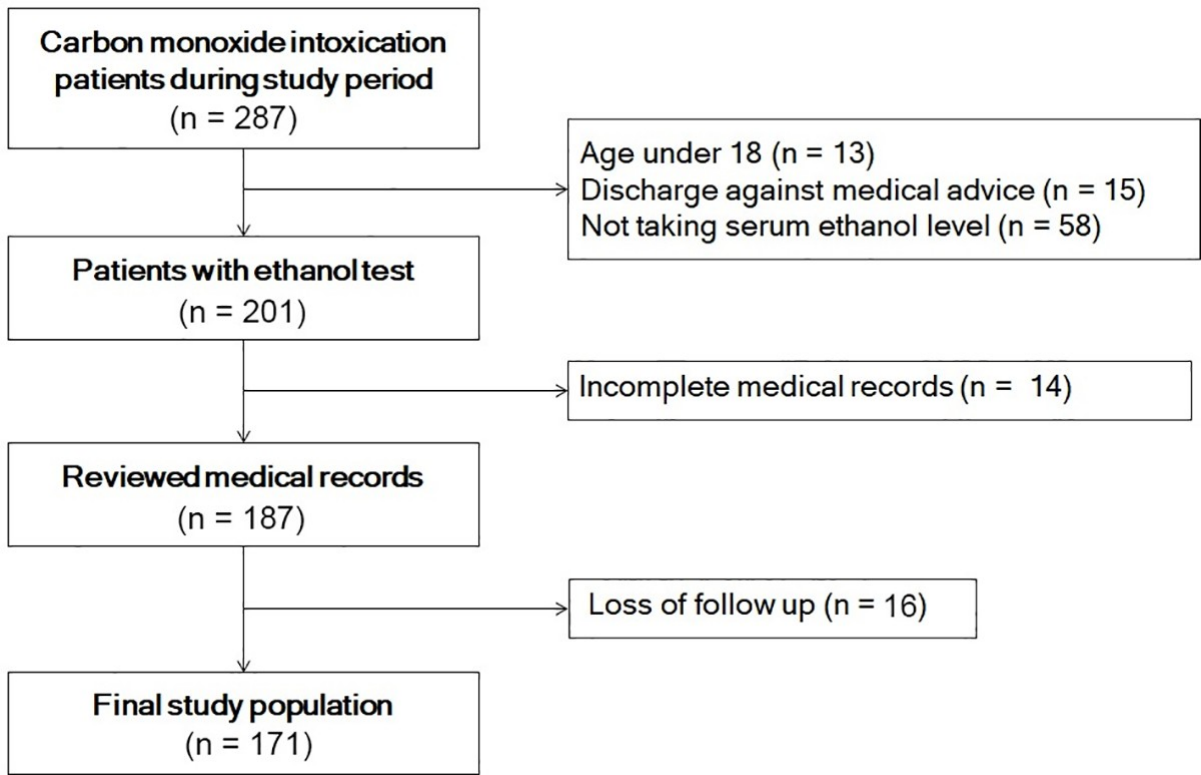
Study population.

During the study period, 287 patients were admitted to the emergency department for CO poisoning. After application of exclusion criteria, 171 patients were included in the study (Fig 1). General characteristics are shown in Table 1. The mean age of the patients was 42.83 ± 14.50 years. The median CO exposure time for all patients was 190 minutes, and 140 patients (81.82%) had intended suicide. Of the 171 patients in this study, 28 (16.37%) developed DNS.

**Table 1:** General characteristics of patients with CO poisoning in this study.

	Total (n = 171)
Age, years	42.83 ± 14.50
Male, n (%)	116 (67.84)
BMI, kg/m^2^	23.05 [20.77, 25.42]
Past history, n (%)	
Hypertension	16 (9.36)
DM	7 (4.09)
MI	4 (2.34)
Ischemic stroke	7 (4.09)
Depression	76 (44.44)
Smoking	96 (61.15)
CO exposure time, min	190 [120, 240]
Intended suicide, n (%)	140 (81.82)
DNS, n (%)	28 (16.37)

Values are expressed as the mean ± standard deviation, median [interquartile range], or number (proportion). BMI, body mass index; DM, diabetes mellitus; MI, myocardial infarction; DNS, delayed neurologic sequelae.

Statistical comparisons of the non-DNS and DNS groups are shown in Table 2. There were no significant differences in age and vital signs between the groups. GCS scores were 15 (10.5–15) in the non-DNS group and 10 (7–15) in the DNS group (p = 0.002). The CO exposure times were 375 min in the DNS group and 190 min in the non-DNS group (p < 0.001). The times from ethanol intake to blood sampling were 172.5 min in the non-DNS group and 288 min in the DNS group. The ethanol levels were 11.01 ± 17.58 mg/dL in the non-DNS group and 1.49 ± 2.63 mg/dL in the DNS group (p < 0.001). However, there were no significant differences in creatinine kinase (p = 0.052), Troponin I (p = 0.237), or COHb (p = 0.605) between the two groups. The MRI lesions of the patients in the DNS group were as follows; 25 patients for periventricular white matter abnormalities, 22 patients for corpus callosum, 14 patients for subcortical U Fibers, 17 patients for external capsule, and 16 patients for internal capsule. Also, the median MMSE score of DNS group was 20 (18–22).

**Table 2:** Comparison of clinical variables between DNS and non-DNS groups.

Variables	Non-DNS (n = 143)	DNS (n = 28)	p-value
Age, years	42.01 ± 13.79	47.04 ± 17.33	0.157
Sex, n (%)			0.122
Female	42 (29.37)	13 (43.43)	
Male	101 (70.63)	15 (53.57)	
BMI, kg/m^2^	23.05 [20.8, 25.51]	22.41 [20.16, 25.39]	0.565
Past history, n (%)			
Hypertension	13 (9.03)	3 (10.71)	0.729
DM	6 (4.20)	1 (3.57)	>0.999
MI	3 (2.1)	1 (3.57)	0.514
Ischemic stroke	3 (2.1)	2 (7.14)	0.403
Depression	64 (44.76)	12 (42.86)	>0.999
Smoking	80 (62.02)	16 (57.14)	0.791
Vital signs			
SBP, mmHg	130 [116, 140]	126 [110, 140]	0.484
DBP, mmHg	80 [71, 90]	80 [70, 90]	0.466
HR, /min	95 [80, 104]	92 [77.5, 102]	0.545
RR, /min	20 [19, 20]	20 [18, 20]	0.268
SaO_2_, %	98 [96, 98.5]	97.5 [94, 98]	0.356
GCS score	15 [10.5, 15]	10 [7, 15]	0.002
CO exposure time, min	190 [105, 190]	375 [191.25, 386.25]	<0.001
Time interval between ethanol intake to sampling, min	172.5 [106.25, 189]	288 [202.5, 412]	0.019
Intended suicide, n (%)	117 (81.82)	23 (82.14)	>0.999
Symptoms, n (%)			
Headache	14 (9.79)	2 (7.14)	>0.999
LOC	40 (27.97)	10 (35.71)	0.551
Nausea/vomiting	2 (1.40)	0 (0)	>0.999
Dizziness	14 (9.79)	1 (3.57)	0.470
Chest pain	4 (2.80)	1 (3.57)	>0.999
Laboratory findings			
WBC, 10^3^/μL	12.43 ± 5.15	13.75 ± 5.46	0.244
Hemoglobin, g/dL	15.14 ± 3.42	14.51 ± 1.56	0.132
Platelets, 10^3^/μL	225.57 ± 91.46	206.04 ± 103.1	0.357
Total protein, g/dL	7.20 ± 0.59	7.22 ± 0.62	0.871
Albumin, g/dL	4.38 ± 0.40	4.35 ± 0.41	0.667
Glucose, mg/dL	128.48 ± 44.24	144.32 ± 55.84	0.166
BUN, mg/dL	14.81 ± 6.56	17.48 ± 6.99	0.071
Creatinine, mg/dL	1.13 ± 0.80	1.11 ± 0.35	0.848
AST, U/L	39.94 ± 46.84	56.11 ± 70.1	0.251
ALT, U/L	33.15 ± 37.94	36.32 ± 41.1	0.707
CK, U/L	815.71 ± 2640.91	2467.96 ± 4008.79	0.052
pH	7.40 ± 0.08	7.37 ± 0.11	0.271
CRP, mg/L	0.89 ± 3.17	1.58 ± 2.77	0.273
Lactate, mmol/L	3.84 ± 3.50	2.34 ± 2.33	0.235
Myoglobin, ng/mL	520.04 ± 1758.62	2861.47 ± 5004.6	0.029
Troponin I, ng/mL	0.27 ± 0.65	0.56 ± 1.08	0.237
CK-MB, ng/mL	12.72 ± 47.07	27.37 ± 39.68	0.098
Ethanol, mg/dL	11.01 ± 17.58	1.49 ± 2.63	<0.001
COHb, %	15.15 ± 13.39	13.70 ± 13.42	0.605
HBOT, n (%)	129 (90.21)	26 (92.86)	>0.99

Values are expressed as the mean ± standard deviation, median [interquartile range], or number (proportion). DNS, delayed neurologic sequelae; BMI, body mass index; DM, diabetes mellitus; MI, myocardial infarction; SBP, systolic blood pressure; DBP, diastolic blood pressure; HR, heart rate; BT, body temperature; RR, respiratory rate; SaO_2_, arterial oxygen saturation; GCS, Glasgow Coma Scale; WBC, white blood cell; BUN, blood urea nitrogen; AST, aspartate aminotransferase; ALT, alanine aminotransferase; CK, creatinine kinase; CRP, C-reactive protein; CK-MB, creatinine kinase myocardial band; LOC, loss of consciousness.

**Fig 2:**
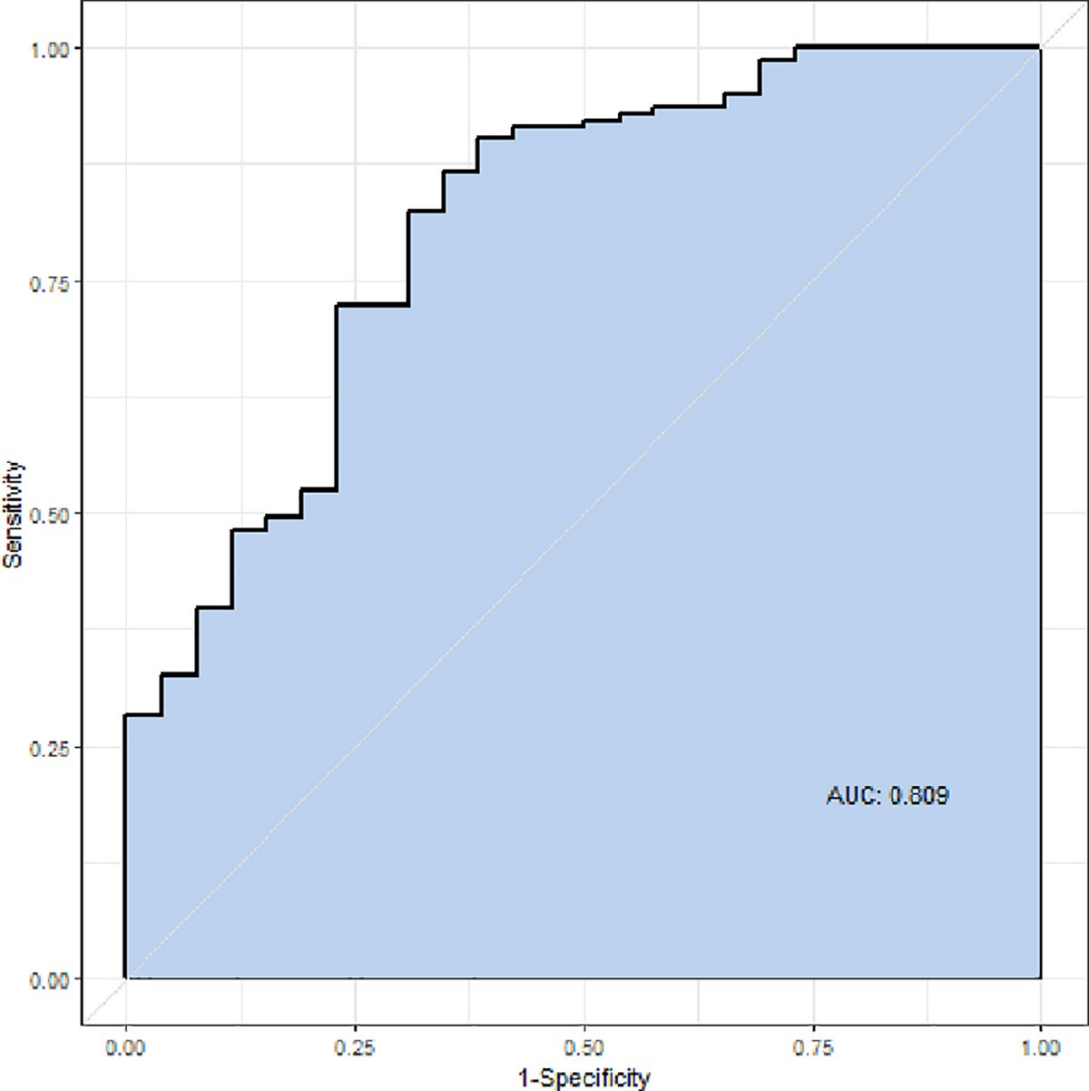
Receiver operating characteristic curve.

The results of univariate and multivariate logistic regression analyses are shown in Table 3. Univariate analysis showed that significant factors associated with DNS were creatinine kinase, CO exposure time, GCS score, and ethanol. Multivariate logistic regression analysis, adjusted for confounding factors, showed that GCS score (odds ratio, 0.770; p < 0.001) and ethanol level (odds ratio, 0.882; p < 0.030) were independent factors for DNS. The area under the receiver operating characteristic curve for the multivariable logistic regression model was 0.809 (confidence interval, 0.713–0.905) (Fig 2).

**Table 3:** Adjusted odds ratios of exploratory variables associated with DNS, following univariate and multivariate logistic regression.

Variables	Univariate		Multivariate	
	Odds ratio (95% confidence interval)	p-value	Odds ratio (95% confidence interval)	p-value
Age, years	1.023 (0.995–1.052)	0.104		
Past History				
Hypertension	1.200 (0.319–4.521)	0.788		
DM	0.846 (0.098–7.304)	0.879		
MI	1.728 (0.173–17.246)	0.641		
Ischemic stroke	3.590 (0.572–22.544)	0.173		
Depression	0.926 (0.409–2.097)	0.853		
BUN, mg/dL	1.054 (0.998–1.112)	0.060		
CK-MB, ng/mL	1.005 (0.997–1.012)	0.198		
Time interval between ethanol intake and sampling, min	1.002 (0.999–1.005)	0.065		
CK, U/L	1.000 (1.000–1.000)	0.019	1.000 (1.000–1.000)	0.203
CO exposure time, min	1.003 (1.001–1.005)	0.002	1.001 (0.999–1.003)	0.336
GCS	0.832 (0.745–0.929)	0.001	0.770 (0.671–0.882)	<0.001
Ethanol, mg/dL	0.904 (0.828–0.988)	0.026	0.882 (0.787–0.988)	0.030

DNS, delayed neurologic sequelae; DM, diabetes mellitus; MI, myocardial infarction BUN, blood urea nitrogen; CK, creatinine kinase; GCS, Glasgow Coma Scale.

## Discussion

We investigated whether ethanol intake had a neuroprotective effect in patients with CO poisoning and could thus influence DNS. Our results showed lower initial GCS score and lower ethanol level were associated with more frequent occurrence of DNS. Thus, ethanol appeared to contribute to the prevention of DNS.

Some studies have reported that N-methyl-D-aspartate (NMDA) receptor inhibition, catecholamine surge control, cerebral edema inhibition by aquaporin-4, and reduced body temperature were beneficial in patients with brain injury [12, 13, 22]. Mechanisms of brain injury caused by CO poisoning include dopamine excess due to hypoxic stress, oxidative stress, lipid peroxidation, and catecholamine stress [10]. CO induces NMDA activation and increases the brain nitrite which damages nerve-cell. Also, lipid peroxidation products induce a lymphocytic immunologic response and microglia activation. By this mechanism, CO causes the neuropathological effects [23]. A possible mechanism for the observed neuroprotective effect of ethanol in brain injury caused by CO poisoning could involve mitigation of sympathetic activity and systemic catecholamine crisis, as well as dopamine suppression in the brain [16, 24]. Also, ethanol has effect on inhibition of NMDA receptor [21], and reduces the infiltration of leukocyte and microglia activation [14, 15]. In this way, ethanol seems to be effective in reducing the neuropathological pathway induced by CO poisoning.

One brain magnetic resonance imaging study reported that ethanol intake was independently associated with a neuroprotective effect against brain lesions [16]. The study’s primary outcome was a brain lesion 5–7 days after exposure to CO, which differed from the primary outcome in our study (i.e., DNS symptoms). DNS can occur regardless of a brain lesion [25]; therefore, it is important to assess the actual symptoms of DNS. Our study confirmed the neuroprotective properties of ethanol by using more practical primary outcomes. Another study concluded that ethanol consumption did not affect the occurrence of DNS symptoms [3]; that study’s primary outcome was the development of neurologic sequelae within 30 days after CO exposure. DNS generally occur after 2 to 40 days of an asymptomatic interval following acute CO poisoning [26]; symptoms reportedly may occur up to several months thereafter [27, 28]. In our study, DNS symptoms were confirmed from repeated telephone interviews after 2 weeks, 1 month, and 3 months; thus, it was possible to capture patients who developed DNS later, which may have contributed to our distinctive findings. In addition, our study data included the time interval between ethanol intake and sampling. Multivariate logistic regression was performed considering these factors, which confirmed that the level of ethanol was associated with the occurrence of DNS, despite correction for these factors. After adjustment for other factors, the initial GCS score was also confirmed to be associated with DNS. Previous studies showed that lower GCS score was associated with worse mortality and neurological outcome in patients with CO poisoning [3, 29]. Our study confirmed that a lower GCS score was associated with greater incidence of DNS.

This study had some limitations. First, it was a prospective study; thus, selection bias may have arisen due to loss to follow up. And the study therefore may have missed patients diagnosed with DNS. Second, we did not assess or control for chemical exposures other than ethanol. As more than 80% of the patients were exposed to CO for suicide purposes, the patients may also have taken other toxic medications for suicide attempt. If patients were exposed to other drugs in addition to ethanol, they may have experienced effects from those drugs. Third, we diagnosed DNS using the neurologic consultation and brain MRI, since a standardized tool for DNS diagnosis was not yet developed [19]. Also, the diagnosis of DNS was made by the one same neurologist. Fourth, our study population is composed largely by suicidal patients who may have psychiatric comorbidities and this could have affected the reliability of their self-reported data. Thus, the result of our study should be interpreted with caution. Fifth, this study was conducted at one university hospital; thus, the results may not be generalizable to other cities or countries. Other hospitals also have distinct protocols for treatment of CO poisoning. Therefore, a prospective large-scale study is needed to confirm these results in other hospitals and other patient populations.

## Conclusions

Higher ethanol level and higher initial GCS score were associated with lower incidence of DNS. Ethanol could have a neuroprotective effect on the occurrence of DNS in CO poisoning patients.

## Supporting information

S1 Fig Delayed neurologic sequelae symptoms checklist (English). (TIF)

S2 Fig Delayed neurologic sequelae symptoms checklist (original). (TIF)
