# Supplementary figures and images for: Effect of ethanol in carbon monoxide poisoning and delayed neurologic sequelae: A prospective observational study
Source: PLoS One. 2021 Jan 11;16(1):e0245265. doi: 10.1371/journal.pone.0245265 (PMC7799805; doi:10.1371/journal.pone.0245265)

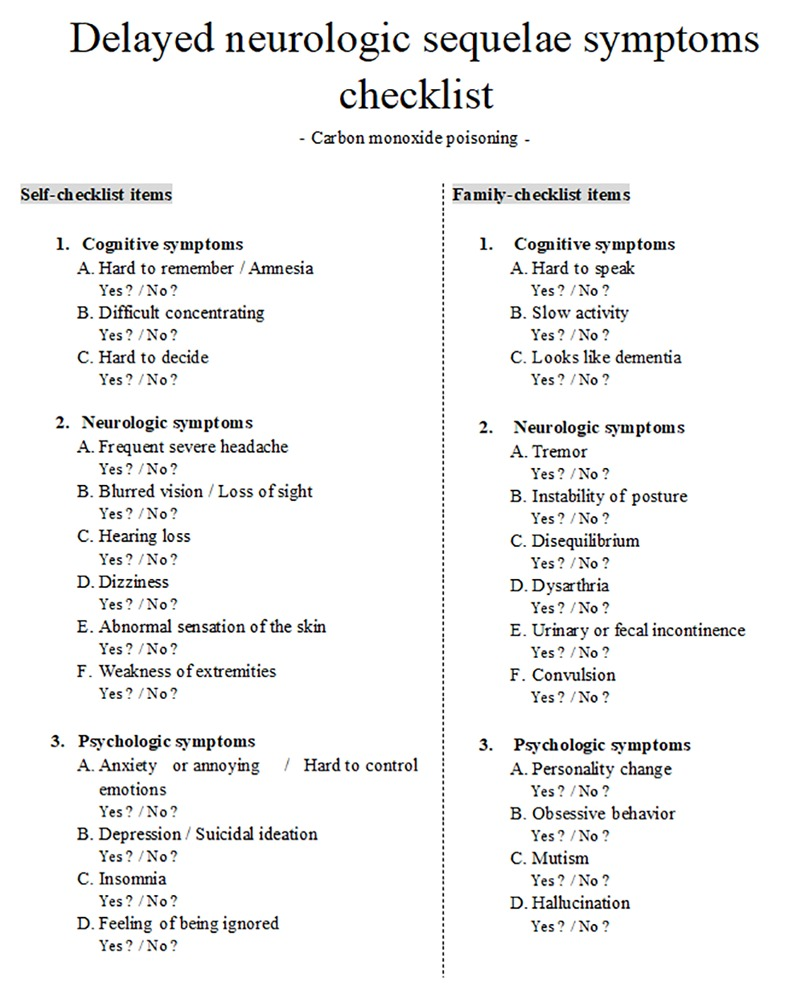

Supplement: S1 Fig — (TIF) [file pone.0245265.s001.tif]

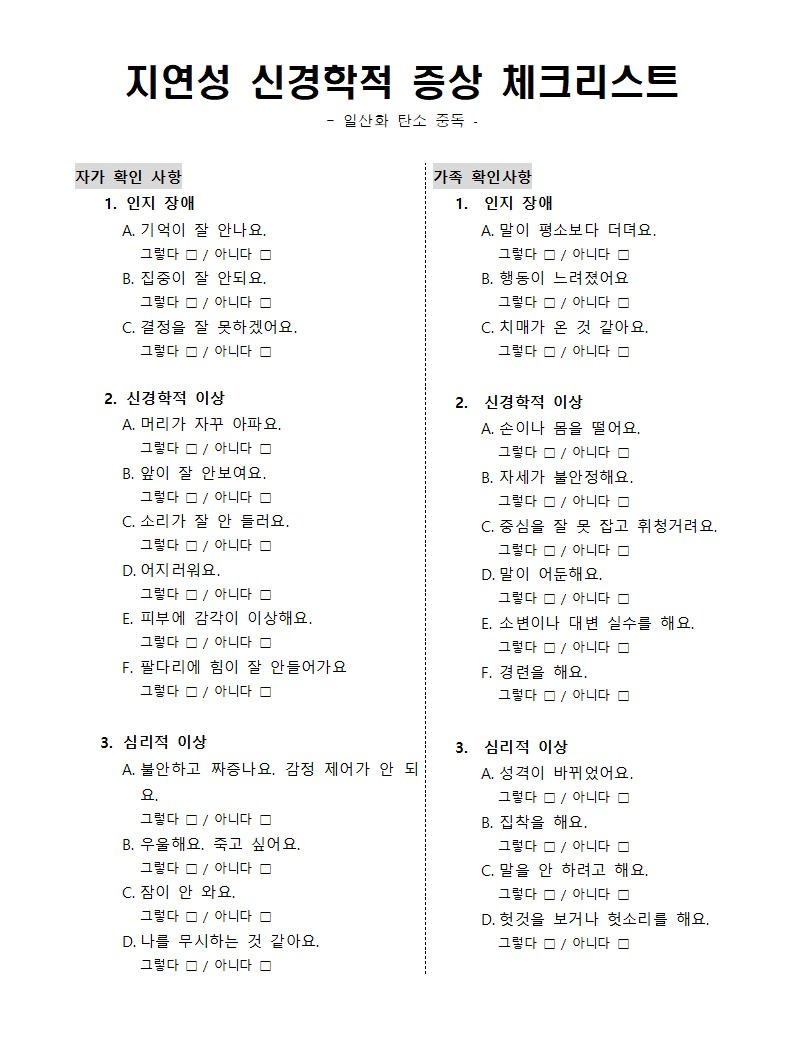

Supplement: S2 Fig — (TIF) [file pone.0245265.s002.tif]
